# Efficacy and Safety of Vedolizumab in Management of Moderate to Severe Ulcerative Colitis: A Systematic Review

**DOI:** 10.7759/cureus.17729

**Published:** 2021-09-05

**Authors:** Renu Bhandari, Opemipo D Ogeyingbo, Roaa Kareem, Mallika Gyawali, Nanditha Venkatesan, Rowan Ahmed, Rinky A Botleroo, Abeer O Elshaikh

**Affiliations:** 1 Internal Medicine/Family Medicine, California Institute of Behavioral Neurosciences & Psychology, Fairfield, USA; 2 Internal Medicine, Manipal College of Medical Sciences, Kaski, NPL; 3 Research, California Institute of Behavioral Neurosciences & Psychology, Fairfield, USA; 4 Public Health, Walden University, Minneapolis, USA; 5 Internal Medicine, Saint James School of Medicine, Park Ridge, USA; 6 Internal Medicine, California Institute of Behavioral Neurosciences & Psychology, Fairfield, USA; 7 Internal Medicine, All India Institute of Medical Sciences, Raipur, IND; 8 Medicine, California Institute of Behavioral Neurosciences & Psychology, Fairfield, USA

**Keywords:** ulcerative colitis (uc), vedolizumab, inflammatory bowel disease, management, biological agent

## Abstract

Ulcerative colitis (UC) is an inflammatory bowel disease and causes inflammation and ulcer of the colon. Vedolizumab is a newer biological agent with an inhibitory effect on α4β7 integrin approved for moderate to severe UC patients. Our study reviewed the clinical response, clinical remission, and mucosal healing of vedolizumab in moderate to severe UC management. Using Preferred Reporting Items for Systematic Reviews and Meta-Analyses (PRISMA) guidelines, we conducted a literature search in PubMed, Google Scholar, and Science Direct, and nine studies were included in the systematic review. At week six, vedolizumab showed a significant clinical response. At week 52, vedolizumab showed significant mucosal healing and clinical remission. The most commonly associated adverse effects are nasopharyngitis, oropharyngeal infection, and gastrointestinal infection. However, additional clinical trials and observational studies with longer follow-ups are required to study the efficacy and safety of the drug.

## Introduction and background

**Figure 1:**
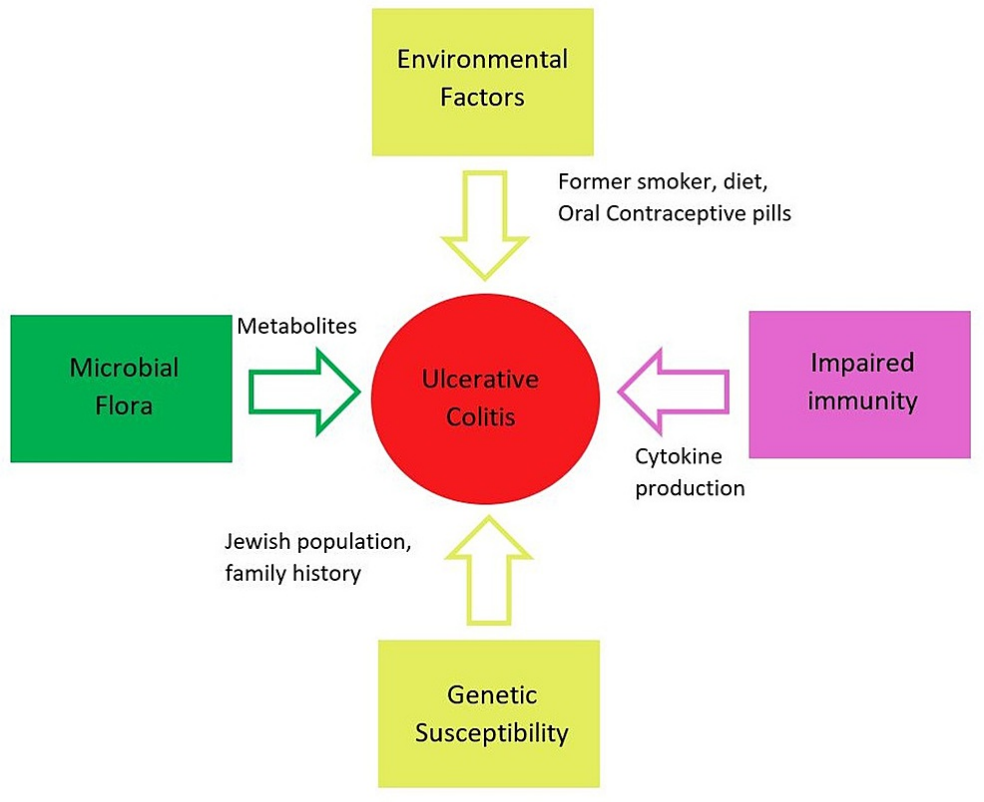
Risk factors of ulcerative colitis

Ulcerative colitis (UC) is a chronic disease that causes diffuse inflammation of the rectal and colonic mucosa [1]. Incidences of UC were reported in Northern Europe (24.3 per 100,000), Canada (19.2 per 100,000), and Australia (17.4 per 100,000) [2]. The prevalence in Asian populations ranges from 5.3 to 63.6 per 100,000 people, whereas in North America, it ranges from 37.5 to 238 per 100,000 people [1]. UC patients usually present with increased frequency of bowel movements, mucus discharge, nocturnal defecation, abdominal pain, diarrhea, tenesmus, slimy stool, and hematochezia [2,3]. In addition, UC is associated with family history, the Jewish population, genetic factors, environmental factors, former smoking history, and drugs like oral contraceptive pills (OCPs) and non-steroidal anti-inflammatory drugs (NSAIDs) [2]. Figure 1 demonstrates the risk factors associated with UC. Endoscopy, colonoscopy, or proctosigmoidoscopy with biopsy is the preferred choice for diagnosis of UC along with clinical signs and symptoms [2,4].

**Figure 2:**
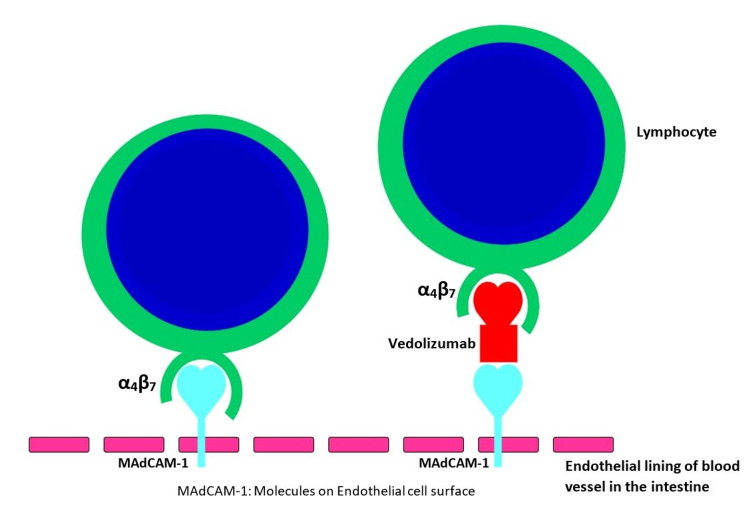
Mechanism of action of vedolizumab Vedolizumab blocks the interaction of the lymphocyte with the ligand MAdCAM-1 present in the vascular endothelial lining of the gut. MAdCAM-1, mucosal addressin cell adhesion molecule-1.

For mild to moderate UC, the first-line treatment is azathioprine, and the patients who do not achieve response are managed with corticosteroid therapy [5]. Moderate to severe cases are treated with thiopurines or biological drugs, or anti-tumor necrosis factor-alpha (TNF-α) medications, such as infliximab, adalimumab, and golimumab [6]. The Food and Drug Administration (FDA) has approved biological therapy like vedolizumab and TNF-α for the treatment of refractory cases in UC [7]. Among biological drugs, infliximab remains the most widely used for ulcerative colitis [7]. A newer biological drug, vedolizumab, was approved for moderate to severe UC cases refractory to standard medications by FDA [2]. In the treatment of failure cases with medical management, colectomy is the preferred method. Patients have experienced many side effects with medications like corticosteroids and TNF-α inhibitors [8]. A study performed by Herrlinger et al. showed about 58% of patients treated with infliximab underwent colectomy [9]. TNF-α inhibitors are associated with severe infections and tuberculosis [10,11]. Similarly, patients with prolonged steroid use experience various systemic side effects [10,11]. Vedolizumab and adalimumab are the humanized monoclonal antibody (HMA) with an inhibitory effect on α4β7 integrin [12]. The α4β7 integrin mediates lymphocyte migration from the circulation across the vascular endothelial barrier into gut-associated secondary lymphoid tissue or the intestinal lamina propria through interaction selectively expressed on the intestine and the interaction blocked by vedolizumab [11]. Figure 2 illustrates the mechanism of action of vedolizumab. Therefore, it has an early clinical response, clinical healing, and endoscopic wound healing in moderate to severe ulcerative colitis due to gut-specific receptor activity and fewer systemic side effects [13].

The complications of UC were toxic megacolon, intestinal perforation, intestinal infarction, myocardial infarction secondary to anemia, and end-stage liver disease due to primary sclerosing cholangitis [1]. In addition, long-standing UC is associated with anxiety, depression, and the development of colorectal carcinoma [14].

This systematic review aims to study the efficacy and safety of vedolizumab in moderate to severe UC patients in early clinical remission, mucosal wound healing, and adverse effect of the medication.

## Review

Methods

Sources of Data and Search Strategy

We used the Preferred Reporting Items for Systematic Reviews and Meta-Analyses (PRISMA) 2020 guidelines. We searched articles by using the databases PubMed, Google Scholar, and Science Direct. The keywords are as follows: ulcerative colitis, inflammatory bowel disease, drug therapy, and treatment. The Medical Subject Headings (MeSH) is the National Library Of Medicine (NLM) control vocabulary thesaurus, specifically used to search articles in PubMed databases. The final search strategy was as follows: (("Colitis, Ulcerative/drug therapy" [majr] OR "Colitis, Ulcerative/mortality" [majr] OR "Colitis, Ulcerative/surgery" [majr] OR "Colitis, Ulcerative/therapy" [majr])) AND "drug therapy" [subheading]. Table 1 illustrates the search strategy.

**Table 1:** Databases and search result

Database	Keywords	Search result
PubMed	As stated above	1,323
Science Direct	(Ulcerative colitis OR inflammatory bowel disease) AND ( drug therapy OR treatment)	885
Google Scholar	Ulcerative colitis AND vedolizumab	94

Inclusion and Exclusion Criteria

We included and selected articles published in the English language, and clinical trials, human studies, adult population, availability of free full text, and articles published between 2017 and May 2021. Studies published in a non-English language, animal studies, review articles, and non-full-text articles were excluded.

Data Extraction and Risk of Bias Assessment

Screening of abstracts and titles was done initially using the Rayyan software (Rayyan Systems Inc., Cambridge, Massachusetts). Then, two authors (Renu and Opemipo) assessed the data independently and screened for all identified studies. A total of 2,302 articles were identified after applying the search criteria, and 2,265 articles remained after removing duplicates. A total of 2,265 articles were screened based on the inclusion and exclusion criteria, and nine papers were selected after the screening process. We used nine randomized clinical trials (RCTs) in our study and assessed the risk of bias using the Cochrane Risk of Bias 2.0 tool.

Results

**Figure 3:**
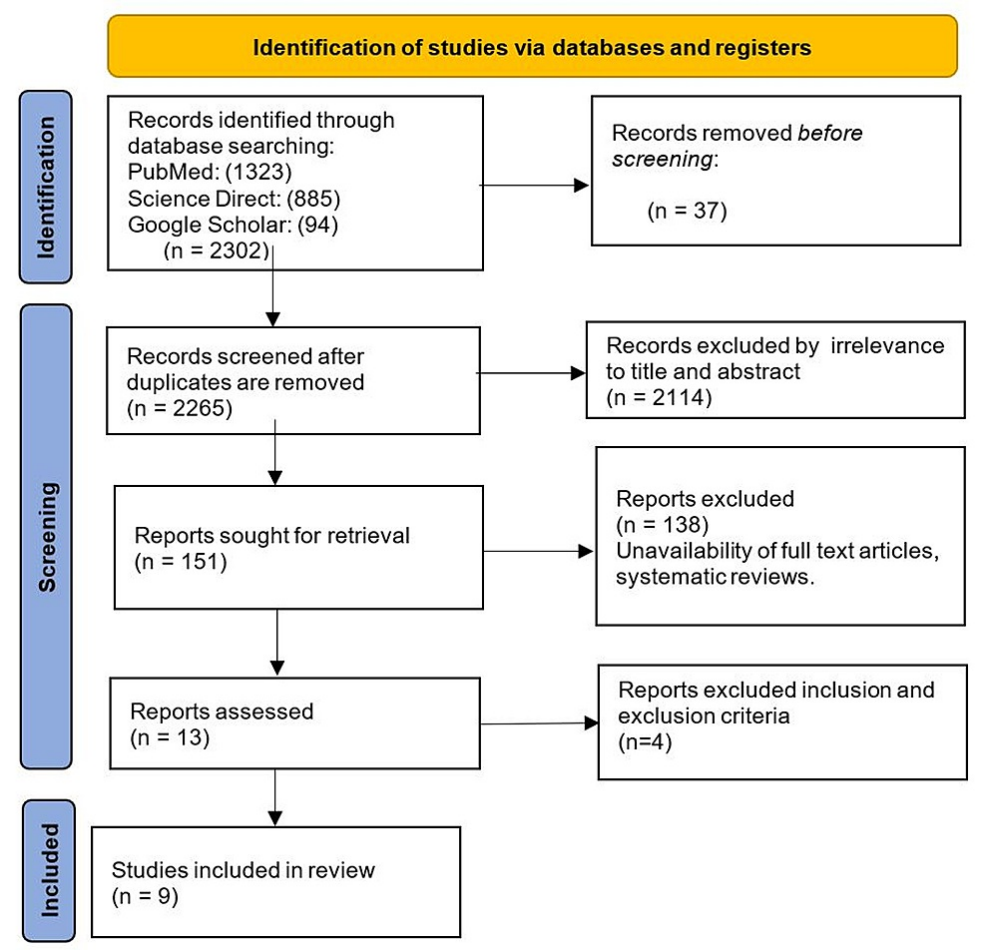
PRISMA flow diagram PRISMA, Preferred Reporting Items for Systematic Reviews and Meta-Analyses.

A total of 2,302 articles were selected from January 2017 to May 2021 using the above search strategy criteria listed in the methods section. The articles were screened based on the title and abstract related to ulcerative colitis (UC) and vedolizumab. After a detailed assessment, we applied inclusion and exclusion criteria and included nine studies, and a complete PRISMA flow diagram was created [15]. Each clinical trial had a similar study design, intervention, and outcome. Figure 3 shows the PRISMA flowchart with the results of this systematic review.

Study Characteristics

Our review includes patients from nine RCT studies. Table 2 shows the characteristics and outcomes of the included studies.

**Table 2:** A tabulated summary of the characteristics of included clinical trials UC, ulcerative colitis; RCT, randomized clinical trial; RDBCT, randomized double-blinded clinical trial; RDBPCT, randomized double-blinded placebo-controlled trial; TNF-α: tumor necrosis factor α; SC, subcutaneous; IV, intravenous.

Authors	Study design	Disease severity and the total number of participants (N)	Intervention	Follow-up	Outcome
Feagan et al. (2017) [16]	RCT phase 3	Moderate to severe UC regardless of TNF-α inhibitors (N: 831)	Vedolizumab and placebo	52 weeks	At week six, vedolizumab-treated patients had a higher clinical response in both TNF-α failure and TNF-α naive patients. At week 52, mucosal healing, clinical remission, and corticosteroid had higher clinical significance in both groups than placebo. >100/1,000 patients in the vedolizumab group had an overall adverse event. Other noted adverse effects are fatigue, nausea, cough, and oropharyngeal infection.
Yajnik et al. (2017) [13]	RDBCT	Moderate to severe UC: 895; Crohn's: 1115	Vedolizumab and placebo	100 weeks	At week six, age <35 had a higher clinical response to the medication than patients >35 in the UC group. At week 52, 35-55 years of age had a statistical difference in clinical remission than age <35 and >55 years of age. An adverse effect like nasopharyngitis and other infections was common in the age group <35 years.
Scribano et al. (2018) [17]	RCT	Moderate to severe UC, TNF-α resistant cases. UC: 800; Crohn's: 747	Vedolizumab and placebo	52 weeks	At week six, vedolizumab-treated patients had a higher statistical difference in clinical response. At week 52, mucosal healing, clinical remission, and corticosteroid-free remission were higher in the vedolizumab-treated patient. Therefore, vedolizumab should be considered the second line for the patient who failed TNF-α therapy. It can also be considered first-line therapy for steroid-dependent and steroid-refractory cases.
Sandborn et al. (2018) [18]	RCT phase 3	Vedolizumab responders were re-randomized (N: 373)	Vedolizumab and placebo	52 weeks	At 52 weeks, 50.6% and 45.5% of patients treated with vedolizumab (four weekly and eight weekly therapy, respectively), and 22.4% treated with placebo had endoscopic improvement. About 28% and 27% of patients treated with vedolizumab (four weekly and eight weekly therapy, respectively), and 8.7% treated with placebo achieved deep remission.
Cohen et al. (2018) [19]	Clinical trial (post-marketing)	Patient with both UC and Crohn's disease (N: 208,050)	Vedolizumab	Four years	Vedolizumab-treated patients in the UC group experienced nasopharyngitis (22%) and gastrointestinal infection (17%), with a 21% infusion reaction rate and 10% overall serious infection. Patients with either Crohn's disease or UC reported 5% opportunistic infection, 19% serious infection, and less than 1% hepatobiliary events.
Motoya et al. (2019) [20]	RDBPCT phase 3	Moderate to severe UC (N: 292)	Vedolizumab and placebo	60 weeks	At week 10, there was no statistical difference in clinical response in the treatment group. At week 60, the clinical remission rate and corticosteroid remission rate were higher in the vedolizumab group, and there was a statistical clinical significance. The rate of nasopharyngitis and adverse events was high in the vedolizumab group compared to the placebo group.
Sands et al. (2019) [21]	RDBCT phase 3	Moderate to severe irrespective of TNF-α (N: 769): vedolizumab (383); adalimumab (386)	Vedolizumab and adalimumab	52 weeks	Vedolizumab was superior to adalimumab in achieving clinical remission and endoscopic improvement but did not achieve corticosteroid-free treatment. The patient's quality of life was better in vedolizumab compared to adalimumab. The overall infection rate in vedolizumab was 62.7%, while in adalimumab it was 69.2%.
Sandborn et al. (2019) [22]	RDBPCT	Moderate to severe UC (N: 216)	Vedolizumab subcutaneous and intravenous formulation and placebo	52 weeks	At week 10, both SC and IV preparation of vedolizumab had a statistically significant clinical response. At week 52, clinical remission and endoscopic improvement with both SC and IV formulation of vedolizumab were higher. The gastrointestinal disorders were 14.2% and 11.1% in SC and IV vedolizumab, respectively, and 32.1% in placebo. The infection rate was 19.8% in SC, 27.8% in IV, and 25% in the placebo group. The rate of serious adverse effects was 9.4% in SC, 13.0% in IV, and 10.7% in placebo. Overall, SC vedolizumab was superior to IV vedolizumab in clinical response, clinical remission, and endoscopic improvement.
Feagan et al. (2019) [23]	RCT placebo-controlled trial	Moderate to severe UC (N: 769)	Vedolizumab and placebo	52 weeks	At week 14, clinical remission was higher in patients with vedolizumab as compared to the placebo group. At week 52, patients who obtained clinical remission at week 14 in the vedolizumab group achieved sustained clinical remission.

Discussion

Therapeutic Benefits of Vedolizumab in Moderate to Severe Ulcerative Colitis

Clinical response is defined as a decrease in Mayo score (MS) from baseline of 30% or more and three or more points, along with either a rectal bleeding subscore of 0 or 1 or a decrease in the rectal bleeding subscore of one point or more [24]. The MS is a combined endoscopic and clinical scale classified into four categories: 0: normal or inactive disease; 1: a mild disease with erythema, decreased vascular patterns, and mild friability; 2: a moderate disease with marked erythema, absence of vascular patterns, friability, and erosions; and 3: a severe disease with spontaneous bleeding and ulceration [25]. Mucosal healing is an absence of friability, blood, erosions, and ulcers in all visualized segments of the gut mucosa [26]. Clinical remission in UC is the disappearance of clinical symptoms, endoscopic appearance, and histologic features; several clinical trials use Mayo score indexing or other indexing methods for the clinical remission of the disease [27].

Feagan et al. conducted a randomized clinical trial (RCT) to study the efficacy of vedolizumab in moderate to severe UC patients irrespective of tumor necrosis factor-α (TNF-α) therapy [16]. The study included 464 patients in TNF-α naïve and 367 patients with inadequate response to TNF-α therapy and followed for 52 weeks [16]. At six weeks, 53.1% of patients naïve to TNF-α and 39% of patients in treatment-resistant to TNF-α therapy achieved clinical response. At 52 weeks, clinical remission was achieved in 46.9% of patients naïve to TNF-α therapy and 36.1% of patients in treatment failure to TNF-α therapy [16].

Similarly, in a study by Scribano et al., in open-label RCT with 800 patients at six weeks, 47.1% of patients in the vedolizumab group and 25.5% in the placebo group achieved clinical response [17]. At 52 weeks, 24% of patients in vedolizumab achieved clinical remission after administering the drug at four weeks intervals. At eight weeks intervals, 20.5% of patients in the vedolizumab group achieved clinical remission [17]. In addition, the vedolizumab group showed higher mucosal healing and higher rates of corticosteroid-free remission [17].

The study by Yajnik et al. included 895 patients in the UC group and conducted a comparative analysis of vedolizumab's efficacy and safety in different age groups less than 35 years, 35 to 55 years, and greater than 55 years [13]. The effectiveness of vedolizumab in achieving clinical response at week six was 51% (<35 years), 47% (35-55 years), and 38% ( >55 years) [13]. In addition, at 52 weeks, clinical remission, mucosal healing, and corticosteroid-free remission were similar in all age groups treated with vedolizumab [13].

The study by Sands et al. conducted RCT in 245 centers in 34 different countries [21]. It included a total of 769 patients to study the difference between vedolizumab and adalimumab for the management of moderate to severe UC patients [21]. At 14 weeks, 31.3% of the vedolizumab group and 22.5% of the adalimumab group achieved clinical remission [21]. At week 52, 39.7% of the vedolizumab group and 27.7% of the adalimumab group achieved endoscopic improvement [21]. However, at the same time, it failed to attain corticosteroid-free remission in the vedolizumab group [21].

Sandborn et al. studied deep remission with vedolizumab in 373 patients with moderate to severely active UC colitis [18]. At six weeks, vedolizumab responders were re-randomized to four-weekly therapy and eight-weekly therapy [18]. At week 52, deep remission was achieved in 8.7% of the placebo group and 28% and 27% of the vedolizumab group with four-weekly and eight-weekly therapy, respectively [18]. Similarly, endoscopic healing in the treatment group was 50.6% and 45.5% at four-weekly and eight-weekly therapy, respectively [18]. Thus, with a higher drug concentration in the blood with four-weekly therapy, vedolizumab achieved a higher rate of deep remission [18].

Feagan et al. conducted post hoc analysis of phase three RCT, placebo-controlled trial to study the sustained clinical remission using MS of less than 2 with no subscore of greater than 1 or rectal bleeding of subscore (RBS) 0 in moderate to severe UC patients [23]. In the study at week 14, a higher proportion of patients treated with vedolizumab obtained clinical remission than the placebo group [23]. Based on MS, 32.7% and 20.1% achieved clinical remission in the vedolizumab and placebo groups, respectively. Similarly, 47.3% and 28.9% achieved clinical remission based on RBS in the vedolizumab and placebo groups, respectively [23]. Sustained clinical remission was higher in the vedolizumab group, who achieved clinical remission at 14 weeks, than in the placebo group [23].

The above-mentioned studies concluded that vedolizumab has a significant clinical response, clinical remission, and mucosal wound healing in moderate to severe UC patients.

In contrast, Motoya et al. conducted RCT double-blinded study in the Japanese population [20]. At week 10, clinical response achieved in the vedolizumab and placebo groups was 39.6% and 32.9%, respectively (p = 0.2722) but there was no statistical significance [20]. At week 60, 56.1% and 31% achieved significant clinical remission in the vedolizumab and the placebo groups, respectively (p = 0.0210) [20]. Therefore, the author concluded that a longer duration of treatment with the drug is required for the response.

Route of Administration of Vedolizumab

Sandborn et al. studied the efficacy and safety of vedolizumab subcutaneous (SC) formulation with UC patients [22]. They conducted a double-blinded RCT in 141 sites in 29 countries and included 216 patients [22]. At 52 weeks, clinical remission was achieved in 46.2%, 42.6%, and 14.3% of patients in SC vedolizumab, intravenous (IV) vedolizumab, and placebo groups, respectively [22]. In addition, SC vedolizumab achieved more remarkable endoscopic improvement than the placebo group [22]. Hence, the author concluded that the SC formulation is better than the IV formulation of vedolizumab because it showed higher clinical remission and can be self-administered [22].

While other included studies used IV formulation, no comparative study was done between SC and IV formulation.

Adverse Effect of Vedolizumab

Cohen et al. studied the safety profile of vedolizumab in UC and Crohn's diseases in four years of the post-marketing survey [19]. Approximately 208,050 patients with UC and Crohn's disease were included in the study [19]. Overall, the risk of serious infection was noted in 10.0% of UC patients, and a gastrointestinal infection was observed in about 17% of UC patients [19]. In both Crohn’s and UC patients, less than 1% malignancy, less than 1% hepatobiliary events, 19% serious infection rate, and 5% opportunistic infection rate were observed [19].

Sands et al. conducted a comparative study between vedolizumab and adalimumab in 245 centers in 34 different countries [21]. The overall infection rate in the vedolizumab was comparatively less than in the adalimumab group [21]. The rate of adverse events in the vedolizumab group and the adalimumab group was 62.7% and 69.2%, respectively [21]. Similarly, the rate of serious adverse events was 11% in the vedolizumab group and 13.7% in the adalimumab group [21].

Sandborn et al. conducted a double-blinded RCT in 141 sites in 29 countries, including 216 patients, to study the efficacy and safety of vedolizumab SC formulation with moderate to severe UC patients [22]. The incidence of injection site reaction in SC vedolizumab was 10.4%, while in IV formulation, the incidence of injection site reaction was 1.9% and 0% in the vedolizumab group and the placebo group, respectively [22]. The gastrointestinal disorders were observed in 14.2% of the SC group, 11.1% of the IV group, and 32.1% of the placebo group [22]. The overall infection rate was 19.8% in the SC group, 27.8% in the IV group, and 25% in the placebo group [22]. And the rate of serious adverse events was 9.4% in the SC group, 13.0% in the IV group, and 10.7% in the placebo group [22].

Motoya et al. conducted a double-blinded RCT study in the Japanese population and included 292 patients [20]. Adverse events in the vedolizumab group were mild to moderate in intensity, and there was no death recorded during the treatment [20].

The study by Yajnik et al. included 895 patients in the UC group and conducted a comparative analysis of vedolizumab's efficacy and safety in different age groups of less than 35 years, 35 to 55 years, and greater than 55 years [13]. The risk of severe infection was lower in the patient population with age greater than 55 years [13]. However, the author believes the difference may be due to the lesser number of patients in that age group [13].

Feagan et al. conducted an RCT to study the efficacy of vedolizumab in moderate to severe UC patients irrespective of TNF-α therapy [16]. The study included 464 patients in TNF-α naïve and 367 patients with inadequate response to TNF-α therapy [16]. The proportion of adverse and serious events in TNF-α naïve were 74% and 9%, respectively, and in TNF-α failure patients, it was 88% and 17%, respectively [16]. The most common noted adverse events in TNF-α failure patients were arthralgia, fatigue, nausea, cough, oropharyngeal pain, and bronchitis [16].

We found few adverse reactions like nasopharyngitis, gastrointestinal infection, and injection site reactions, and no significant side effects like malignancy, leukoencephalopathy, and tuberculosis with the drug from our analysis. Few side effects reported may be due to the gut specificity of the drug. Hence, vedolizumab is well-tolerated and safe in patients with moderate to severe UC.

Limitations

Our article has some limitations; only the articles published in the English language, with the availability of free full text and clinical trials were included. In addition to this, patients in these included studies were only followed for 52 weeks and did not look for the relapse of the disease. Therefore, more RCTs with longer follow-ups are required to study more about the efficacy and safety profile of the drug.

## Conclusions

The main objective of this article is to study the efficacy and safety of vedolizumab in the management of moderate to severe UC patients. Most of the included studies showed a significant clinical response and clinical remission of the disease. Additionally, vedolizumab is well-tolerated with fewer side effects and is safe to use in UC patients. In comparison to IV formulation, the SC formulation of vedolizumab was more effective, and the four-weekly therapy was better compared to the eight-weekly therapy. Comparative studies of vedolizumab with other biological agents will be beneficial and worth exploring as this drug is gut selective and efficient with lesser side effects.
